# Supplementary material for: Time-series transcriptome analysis identified differentially expressed genes in broiler chicken infected with mixed Eimeria species
Source: Front Genet. 2022 Aug 8;13:886781. doi: 10.3389/fgene.2022.886781 (PMC9393255; doi:10.3389/fgene.2022.886781)
Supplement: Supplementary file 2 [file DataSheet1.ZIP › 7dpi_KEGG.Gsea.1625115961950/KEGG_EPITHELIAL_CELL_SIGNALING_IN_HELICOBACTER_PYLORI_INFECTION.html]

Details for gene set KEGG\_EPITHELIAL\_CELL\_SIGNALING\_IN\_HELICOBACTER\_PYLORI\_INFECTION[GSEA]

|  || Dataset | TMM\_7dpi\_gct\_format\_7dpi\_gct\_format.Class\_4dpi.cls #PC\_versus\_NC.Class\_4dpi.cls #PC\_versus\_NC\_repos |
| Phenotype | Class\_4dpi.cls#PC\_versus\_NC\_repos |
| Upregulated in class | 1 |
| GeneSet | KEGG\_EPITHELIAL\_CELL\_SIGNALING\_IN\_HELICOBACTER\_PYLORI\_INFECTION |
| Enrichment Score (ES) | 0.47662526 |
| Normalized Enrichment Score (NES) | 2.2040377 |
| Nominal p-value | 0.0 |
| FDR q-value | 9.861911E-4 |
| FWER p-Value | 0.0048 |
Table: GSEA Results Summary

  

Fig 1: Enrichment plot: KEGG\_EPITHELIAL\_CELL\_SIGNALING\_IN\_HELICOBACTER\_PYLORI\_INFECTION      
 Profile of the Running ES Score & Positions of GeneSet Members on the Rank Ordered List

  

| SYMBOL | TITLE | RANK IN GENE LIST | RANK METRIC SCORE | RUNNING ES | CORE ENRICHMENT || 1 | ATP6V1C2 | na | 4 | 2.128 | 0.0553 | Yes |
| 2 | HBEGF | na | 12 | 1.793 | 0.1016 | Yes |
| 3 | ATP6V0A4 | na | 19 | 1.686 | 0.1452 | Yes |
| 4 | ATP6AP1 | na | 21 | 1.657 | 0.1884 | Yes |
| 5 | ATP6V0D2 | na | 24 | 1.636 | 0.2310 | Yes |
| 6 | ATP6V0C | na | 30 | 1.603 | 0.2725 | Yes |
| 7 | ATP6V1D | na | 46 | 1.458 | 0.3094 | Yes |
| 8 | ATP6V1E1 | na | 61 | 1.395 | 0.3447 | Yes |
| 9 | ATP6V0E1 | na | 72 | 1.354 | 0.3792 | Yes |
| 10 | ATP6V1H | na | 80 | 1.313 | 0.4129 | Yes |
| 11 | ATP6V0B | na | 100 | 1.252 | 0.4441 | Yes |
| 12 | ATP6V1G3 | na | 102 | 1.247 | 0.4766 | Yes |
| 13 | MAPK11 | na | 678 | 0.646 | 0.4454 | No |
| 14 | CXCR1 | na | 701 | 0.635 | 0.4602 | No |
| 15 | ATP6V1G1 | na | 2444 | 0.199 | 0.3196 | No |
| 16 | NFKBIA | na | 2532 | 0.187 | 0.3172 | No |
| 17 | JAM2 | na | 2750 | 0.157 | 0.3032 | No |
| 18 | RAC1 | na | 2998 | 0.126 | 0.2858 | No |
| 19 | MET | na | 3037 | 0.122 | 0.2858 | No |
| 20 | GIT1 | na | 3387 | 0.075 | 0.2586 | No |
| 21 | CSK | na | 3472 | 0.066 | 0.2533 | No |
| 22 | ADAM17 | na | 3490 | 0.064 | 0.2535 | No |
| 23 | TCIRG1 | na | 3866 | 0.020 | 0.2226 | No |
| 24 | NOD1 | na | 3973 | 0.008 | 0.2140 | No |
| 25 | ATP6V1C1 | na | 4112 | -0.007 | 0.2026 | No |
| 26 | MAP2K4 | na | 4132 | -0.010 | 0.2013 | No |
| 27 | JAM3 | na | 4165 | -0.013 | 0.1989 | No |
| 28 | JUN | na | 4404 | -0.040 | 0.1801 | No |
| 29 | ATP6V0A1 | na | 4522 | -0.052 | 0.1717 | No |
| 30 | MAPK13 | na | 5231 | -0.124 | 0.1157 | No |
| 31 | ATP6V0D1 | na | 5522 | -0.151 | 0.0954 | No |
| 32 | CDC42 | na | 5837 | -0.184 | 0.0739 | No |
| 33 | ATP6V1A | na | 5917 | -0.192 | 0.0723 | No |
| 34 | ATP6V1B2 | na | 5973 | -0.199 | 0.0729 | No |
| 35 | PLCG1 | na | 6241 | -0.228 | 0.0565 | No |
| 36 | RELA | na | 6283 | -0.233 | 0.0592 | No |
| 37 | SRC | na | 6405 | -0.244 | 0.0554 | No |
| 38 | CASP3 | na | 6448 | -0.248 | 0.0584 | No |
| 39 | MAPK8 | na | 6464 | -0.250 | 0.0637 | No |
| 40 | ATP6V0E2 | na | 6836 | -0.290 | 0.0402 | No |
| 41 | PTPRZ1 | na | 7010 | -0.309 | 0.0339 | No |
| 42 | ADAM10 | na | 7357 | -0.347 | 0.0140 | No |
| 43 | MAPK12 | na | 7612 | -0.373 | 0.0025 | No |
| 44 | MAPK9 | na | 7648 | -0.377 | 0.0094 | No |
| 45 | IKBKB | na | 8465 | -0.479 | -0.0463 | No |
| 46 | MAPK14 | na | 8502 | -0.484 | -0.0367 | No |
| 47 | PAK1 | na | 8943 | -0.541 | -0.0594 | No |
| 48 | ATP6V0A2 | na | 9318 | -0.602 | -0.0749 | No |
| 49 | NFKB1 | na | 9346 | -0.606 | -0.0613 | No |
| 50 | CHUK | na | 9554 | -0.639 | -0.0620 | No |
| 51 | TJP1 | na | 10016 | -0.715 | -0.0818 | No |
| 52 | PTPN11 | na | 10560 | -0.822 | -0.1058 | No |
| 53 | MAP3K14 | na | 10581 | -0.829 | -0.0858 | No |
| 54 | PLCG2 | na | 10697 | -0.853 | -0.0731 | No |
| 55 | CCL5 | na | 10974 | -0.929 | -0.0719 | No |
| 56 | LYN | na | 11314 | -1.043 | -0.0730 | No |
| 57 | EGFR | na | 11415 | -1.085 | -0.0530 | No |
| 58 | IGSF5 | na | 11653 | -1.202 | -0.0414 | No |
| 59 | MAPK10 | na | 12008 | -2.724 | 0.0002 | No |
Table: GSEA details [plain text format]

  

Fig 2: KEGG\_EPITHELIAL\_CELL\_SIGNALING\_IN\_HELICOBACTER\_PYLORI\_INFECTION      
 Blue-Pink O' Gram in the Space of the Analyzed GeneSet

  

Fig 3: KEGG\_EPITHELIAL\_CELL\_SIGNALING\_IN\_HELICOBACTER\_PYLORI\_INFECTION: Random ES distribution      
 Gene set null distribution of ES for **KEGG\_EPITHELIAL\_CELL\_SIGNALING\_IN\_HELICOBACTER\_PYLORI\_INFECTION**
